# Accessing needle exchange services in disasters for remote areas of Aotearoa New Zealand

**DOI:** 10.1186/s12954-022-00709-2

**Published:** 2022-12-22

**Authors:** Anne Rijnink, Denise Blake, Shiloh Groot, Chris Brough

**Affiliations:** 1 School of Psychology, Massey University, Wellington, Aotearoa New Zealand; 2 School of Health, Te Herenga Waka-Victoria University of Wellington, Wellington, Aotearoa New Zealand; 3 School of Psychology, University of Auckland, Auckland, Aotearoa New Zealand; 4 NICHE, Nelson, Aotearoa New Zealand

**Keywords:** PWID, Health care, Access, Rural regions, Disasters, Preparedness, Qualitative research

## Abstract

**Background:**

Needle Exchange Programme (NEP) mobile outreach services in Aotearoa New Zealand distribute injecting equipment to people who inject drugs (PWID) living in remote regions. In disasters, continued access to such services is imperative for the health and wellbeing of PWID. Disasters can compound existing inequities, particularly in regions characterised by poor or limited infrastructure, smaller populations, and challenging socioeconomic conditions. To gain insight into the barriers that prevent access to NEP harm-reduction services and understand the needs of PWID prior to and during disasters, this study foregrounds the voices of PWID based on the West Coast of the South Island, Aotearoa New Zealand.

**Methods:**

This qualitative study applied an interpretive phenomenological analysis approach, where 14 PWID and one key NEP staff member took part in semi-structured interviews. The interviews provided the opportunity for participants to share their experiences and perspectives about accessing sterile drug-injecting equipment during disasters, including the four-week COVID-19 Level 4 lockdown in March 2020. In total five superordinate and 14 subordinate themes were identified from the interveiws.

**Results:**

This study focuses on four of the key themes that impacted accessibility to NEP services: infrastructural hazards and equipment costs; social capital and practical support from peers and key contact networks; social stigma in public locations, including NEP-based pharmacies and emergency centres; and potential solutions to NEP equipment accessibility as frequently suggested by participants.

**Conclusions:**

Access to NEP services is essential during natural hazard and human-generated disasters, as such NEP mobile outreach services and disaster resilience efforts should focus on maintaining service continuity for PWID during adverse times. This study champions a needs-based, stigma free approach to inclusive harm-reduction and emergency management practices for groups with specific needs in a disaster context.

## Background

Continued access to a Needle Exchange Programme (NEP) is imperative for People Who Inject Drugs (PWID)^1^, ^2^, particularly during extraordinary times such as when natural hazards or human-generated disasters occur. The NEP is a healthcare service that provides a pragmatic and compassionate approach to reducing drug-injecting harm for individuals and communities [1, 2]. NEPs aim to meet people where they are in their lives by recognising that abstinence from drugs can be an unrealistic goal for many [3]. Consequently, it is appropriate to provide people who inject substances with sterile injecting equipment. Ready access to products, including sterile needles and syringes, decreases the likelihood that these products will be reused or shared, which in turn reduces the risk of contracting blood-borne virus (HIV/AIDs, HBV and HCV) [1, 4] or other physical issues, like abscesses [5]. Moreover, injection drug use is subject to stigma [6] that can foster feelings of shame, hopelessness and isolation in PWID. NEPs are staffed by trained peers based on the philosophy that people with lived experience of drug use are likely to provide a trusted point of contact and a place to feel safe, free of coercion, discrimination, and stigma [2, 7].

While NEP services are known to be successful at preventing social and health harms, access to these services can be problematic [8, 9, 10, 11]. International literature documents how PWID face a range of barriers to healthcare in ordinary times, including access to doctor’s clinics [12, 13], HCV treatment centres [14, 15, 16], and NEP [8, 9, 10, 11]. Commonly cited healthcare barriers include cost [13, 17, 18], travelling distances, operational hours [11, 13, 18, 19], and stigma [14, 17, 20, 21]. What is less known is how PWID are enabled and constrained in their access to the NEP during disasters, including the COVID-19 pandemic. To further this knowledge, this study explores the experiences of PWID who rely on a NEP mobile outreach service on the West Coast of the South Island in Aotearoa New Zealand.

### The West Coast NEP mobile outreach service

The West Coast NEP mobile outreach service was established in 2002 and is one of two mobile outreach needle exchange services in Aotearoa New Zealand [22]. The mobile outreach service distributes sterile drug-injecting products to people who reside in remote areas, including the West Coast. PWID living remotely or too far from static NEP exchanges, can also now access equipment through the COVID-19 inspired online NEP store, where products can be ordered and delivered via a courier [23]. Besides this, PWID on the West Coast can also acquire sterile drug-injecting products through key contact networks, who are peers that reside in remote communities that are given NEP equipment to distribute amongst the local drug-injecting community [24].

**Fig. 1:**
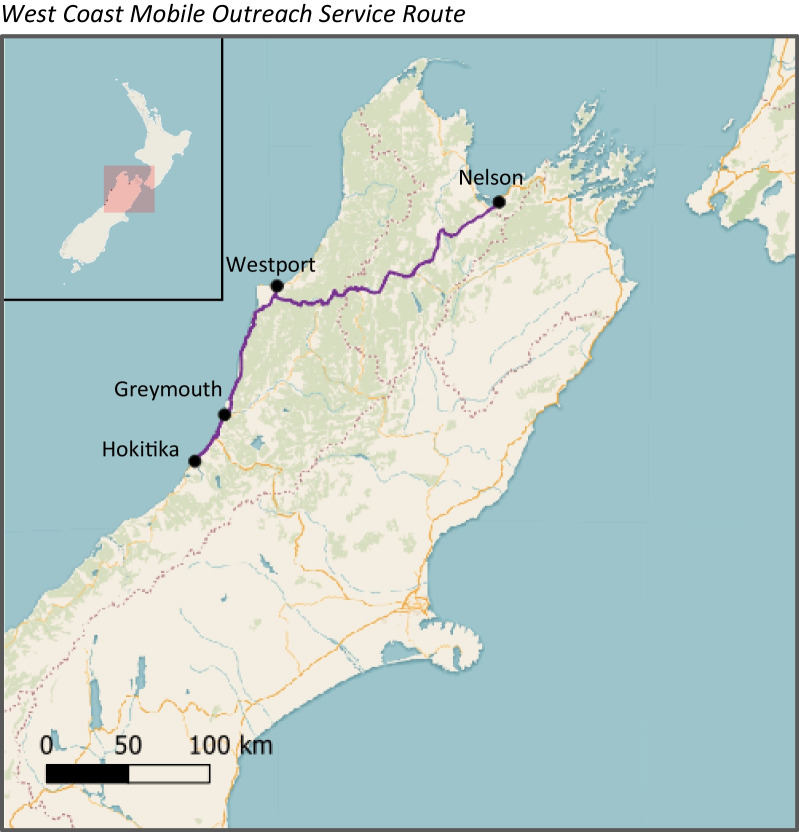
West Coast Mobile Outreach Service Route. This is a map of the South Island of Aotearoa New Zealand showing the West Coast Mobile Outreach Service Route which follows the State Highway 6 from Nelson to Westport, to Greymouth, and then to Hokitika

The NEP mobile outreach service was managed by a key Nelson-based NEP staff member who would drive the 3.5-h trip to the West Coast via State Highway 6 monthly. This outreach service caters to the approximately 40 PWID living in Greymouth, Hokitika, and Westport (small townships along the West Coast of the South Island of Aotearoa New Zealand) (see Fig. 1).

### Disaster risks

The success of the NEP outreach service is threatened by natural hazard risks [25, 26]. For instance, the West Coast region lies adjacent to Aotearoa New Zealand’s “big-risk” Alpine Fault, which has an estimated 85% chance of causing a powerful earthquake (e.g. magnitude 8 and higher) in the next 100 years [27] that could impede all inter-district travel for at least one week [28]. Another high-risk hazard on the West Coast is flooding, particularly in Westport, which is surrounded by two large bodies of water: the Tasman Sea and the Buller River. The Westport township sits on low-lying ground and has limited flood protection [29]. In recent years, there have been several major floods in this region: two significant flooding events in February 2018 [30], and a July 2021 flood that damaged over 800 properties and caused the evacuation of 2000 of Westport's 4500 residents [31, 32]. The 2021 flood prevented road access in and out of Westport for several days [31].

A further risk to NEP service accessibility are human-generated disasters, such as the COVID-19 pandemic. Between March and April 2020, the Aotearoa New Zealand government controlled the spread of COVID-19 through a nation-wide lockdown, meaning physical distancing and travel bans were in place [33]. These unprecedented restrictions caused disruption to daily life, with research demonstrating that depression and anxiety significantly exceeded population norms [34]. International studies [35, 36, 37, 38, 39, 40] describe how people using substances were likely to be disproportionately susceptible to the COVID-19 virus and its stressors. For instance, Farhoudian and colleagues [36] argued that having minoritised and stigmatised statuses and existing health conditions, likely increased vulnerability to the effects of COVID-19.

The NEP was regarded as an essential service during COVID-19 Level 4 lockdown in Aotearoa New Zealand, which meant that NEP could continue operating, alongside other health and emergency services. However, the monthly West Coast mobile outreach service was delayed (by management) for a week in the first stage of the Level 4 lockdown. This meant that there was one week where PWID were unable to access equipment via the outreach service.

Few studies have mapped the specific risks surrounding access to NEP products in disaster settings; however, research by Blake et al. [41], found that people receiving Opioid Substitution Treatment (OST), another harm-reduction life-sustaining treatment for PWID, knew that stigma would position them as undeserving of help and they expected poor treatment by people in authority during emergencies. Blake et al. [42] also noted that Aotearoa New Zealand disaster preparedness messaging via government documents and preparedness websites mostly privilege agency to those with resources, and deny it to other more minoritised groups.

Broader disaster-based literature has illustrated how disasters can compound existing social and political vulnerabilities for minoritised groups due to a lack of recognition, representation, and social security [43, 44, 45, 46, 47, 48, 49, 50, 51]. Another explanation for the increased disadvantage is that people who endure hardship have less socioeconomic resilience or a lessened ability to respond to, and recover from, disaster harm [52, 53]. Structural disadvantage can mean people lack the ability to prepare for a disasters as household emergency plans typically require storing extra resources (food, water, torches, small tent, hygiene items, petrol and medications) and having insurance to replace destroyed assets [54]. Furthermore, people who are minoritised often live in substandard or insecure housing that can be more prone to damage from natural hazards, such as earthquakes [55].

Rural areas in Aotearoa New Zealand have additional challenges for risk management, emergency preparedness, and resilience-building more generally as, for instance, limited infrastructural and technological capabilities than urban areas could mean people living in remote areas are unable to fully benefit from the resources made available after a disaster [56, 57]. Moreover, remote living in Aotearoa New Zealand can hinder people’s ability to reach or be reached by emergency services [58]. This research seeks to add to disaster and healthcare literature by documenting what enables and constrains access to NEP services for rurally based PWID using the NEP mobile outreach service.

## Method

### Design

This study engaged an interpretive phenomenological analysis (IPA) framework and method [59, 60, 61]. Based on phenomenological theory and assumptions, IPA has an idiographic focus, in that it offers insights into how a person, in a given context, makes sense of a phenomenon through open-form inquiry [62, 59, 61]. IPA enabled us to explore the psychological and social worlds of PWID. For instance, IPA was used to investigate the role of stigma, as a way to make sense of how PWID are excluded from disaster risk reduction initiates. Stigma prevents full social acceptance, causing PWID to be devalued, and rejected because of their discredited health condition [63, 64, 65]. Akin to other contexts, in a disaster stigma can be experienced at the level of the self (internalised stigma), social (between people) and on a structural dimension (in the form of institutional stigma) [7, 67, 68].

### Procedure

This study was community-driven, whereby a key community member emailed the second author to discuss concerns about access to NEP products following flooding on the West Coast. They wanted to ensure there were emergency management plans in place. Consultation occurred with key community members, especially the key staff member from the Nelson NEP, at all stages of the research. The research design, methods, and practical applicability were also determined by the community of interest, with relational ethics [69], unconditional positive regard and respect always practised. Ethical approval was provided by the Massey University Human Ethics Committee (Northern, Application NOR 20/27).

### Participant recruitment

Participants were recruited by the key Nelson-based NEP staff member who travelled to the West Coast. These participants were approached after having expressed a prior interest in, and concern about, accessing drug-injecting equipment during floods with the NEP staff member. Fourteen participants took part in interviews, including 13 PWID from across the West Coast region, and the key Nelson-based NEP staff member. Participants were between 30 and 51 years old. There were six people who identified as female and eight identified as male; all participants had received Opioid Substitution Treatment (OST) at some time. Seven semi-structured interviews were held face-to-face, and 7 over the phone. All interviews were audio-recorded and lasted approximately an hour. Interviews were transcribed verbatim and anonymised. After each interview, participants were given a supermarket voucher (worth $40.00NZD) in recognition of their time.

### Data analysis

Following Smith and Shinebourne [61], the first author applied an IPA-style interpretation, that was iterative and continuous. The interpretation process involved reading transcripts and making early notations of broad interpretations about the participants experience. In subsequent read-throughs, associations between transcripts and notes were transformed into higher-level abstractions and assigned conceptual labels. These were eventually reduced to a final list of superordinate (higher-level) and subordinate themes (lower-level). These findings were discussed with the other researchers throughout the process. As part of the interpretation, to achieve the balancing double hermeneutic process [59], reflexivity ensured that the research findings prioritised new data over any preconceptions and biases [70].

## Results

Four superordinate themes were identified. From these stem several subordinate themes. The first superordinate theme discusses elements that could hinder outreach accessibility in a disaster, including infrastructure and equipment costs. The second superordinate theme identifies the importance of peers, and includes social capital, practical support and key contact networks. The third superordinate covers social stigma, including fear of stigma from managers at emergency centres. The last superordinate theme explores the most frequently mentioned solutions to NEP service disruptions during disasters. These solutions are hypothetical and additional to current services (such as the online NEP store, courier delivery and a key contact system). All superordinate themes and their subthemes are substantiated by excerpts taken from participant narratives.

### Infrastructure and equipment costs

#### Distance and road vulnerability challenges

Participants in this study were aware of the risks associated with disasters. Kyle^3^, for instance, recognises that he might not be able to access the mobile outreach service due to critical infrastructure failures, specifically blocked road networks. Kyle attributed this to living remotely:

We’re so remote on the West Coast […]. We’re cut off in the event of a disaster. Just a couple of roads could block, and nothing (injecting products/OST) could come in. If we had a big earthquake […], the only supplies (that would go in) are essential medical supplies, food, and water.

In his wider narrative, Kyle mentions that Westport is surrounded by water, and roads vulnerable to erosion could easily prevent access to the NEP mobile outreach service. Kyle discusses how essential health gear (like NEP products or OST medications) will likely not be prioritised for PWID in emergencies, unlike other medical supplies. David is also aware of accessibility issues people in smaller towns face. He compares accessibility in remote areas and cities:

Here in the small town, you can’t access what you want readily as in the city. You know, you have lots of options in the city, but here people just sort of bite the bullet. Oh, they do the rounds, see what they can do, but it’s a small cliquey little town.

As David suggests, remote areas do not have static NEP exchanges and limited local options to source equipment (e.g. NEP-based pharmacies, key contacts), which expose people to harm if they need to reuse or share injecting equipment. Participants’ discussions around infrastructure vulnerabilities from natural hazards led them to identify other disaster preparedness actions, such as storing NEP products in advance, as Kyle’s quote demonstrates:

I have enough [NEP products] for the month plus another two weeks in case something happens […]. I do it consciously because I live remotely, and there’s always something that could happen.

Kyle’s foresight in preparing for a “happening” outside of the everyday was an approach based on living remotely. He was one of the few in this study who had prepared for an emergency by storing general disaster survival essentials (water, food, blankets) as suggested by the New Zealand National Emergency Management Agency (NEMA) [71]. However, lower overall preparedness rates were not unexpected, given that, on average, only 24% of the Aotearoa New Zealand population have fully prepared for an emergency [72]. Overall, these findings highlight how critical infrastructure vulnerabilities, such as roading blockages, are recognised by the PWID in this study as potential barriers to accessing safe injecting equipment. These accessibility problems emphasise the importance of local emergency planning that caters to *all* community needs.

#### Cost of products

As other scholars argue [43, 44, 45, 46, 47, 48, 49, 50, 51], disasters can exacerbate existing vulnerabilities for minoritised people. This includes financial hardships, which, in turn, perpetuates and reinforces poor health outcomes. Harriet spoke about the cost of drug-injecting equipment as a barrier in everyday life because she and her partner are unable to afford to purchase items and, at times, had to reuse their needles and syringes:

When the [NEP mobile outreach service] comes, all we get is four butterflies and four barrels, and I have to reuse them until [the staff member] is here next cos that’s all we can afford to do.

We can presume from Harriet’s excerpt that she has reused the same equipment numerous times as it is approximately one month between mobile outreach visits to the West Coast. Her action is concerning as reusing drug-injecting equipment carries risk. For example, butterflies are needles that are used when people have trouble finding veins or when there are larger amounts of liquid being injected. Reusing butterflies (or any needle) can cause bacteria to enter the body and cause other harm [5]. We can also presume that cost will be a problem following a disaster.

During the Level 4 COVID-19 lockdown period, temporary Income Relief Payments by Work and Income [73] and other forms of social support increased, including food parcels [74] and the provision of ‘safe’ housing [73]. An online NEP store enabled people to make confidential orders that were delivered by courier to minimise human contact during the 2020 COVID-19 Level 4 lockdown [23]. However, it is not uncommon for people living with socioeconomic hardship or in remote areas to not own a smartphone or be computer literate [56]. Any absence of being online negates the utility of online options for purchasing goods or any other internet-based emergency management solutions [75]. All participants reported underutilising the online NEP service due to limited cell phone coverage or minimal internet use, for instance, Christy declares, “I don’t do online […] I would have to be able to ring up and order”. For Christy, a phone order was best. Additionally, the online courier delivery incurs a fee, which some PWID are unable to cover due to their limited budget. As a way to counteract this, according to the mobile outreach staff, “donation money” (spare money donated by other PWID) was used during that time to cover the costs of sterile drug-injecting equipment for those who needed help, likely reducing any financial strain:

Key contacts were stocked up massively with all the free and spare non-free stock I could find…. During COVID-19, I’ve made sure that the Needle Exchange donations were used up, plus a couple of other NEP staff also donated equipment and cash. (NEP staff)

Providing additional financial and social incentives during a disaster goes some way towards improving resiliency for communities that have material disadvantages. It also demonstrates the importance of peer-based support.

### Peers

#### Social capital and practical support

Peer-based services, like NEPs, represent a form of social capital in action where the networks and relationships between people and communities can enhance wellbeing during and following disasters. Being peer-based meant that the mobile outreach staff could take either an active role (e.g. offering PWID the opportunity of a courier order) or a passive role (e.g. stocking up key contacts with additional free stock to distribute) in anticipation of a disaster. Ultimately, this foresight was instrumental in preventing product shortages on the West Coast throughout the 2020 lockdown period. The following quote also reflects the importance of committed and adaptable service providers that ensure product accessibility for PWIDS, no matter what:

I’m really okay with anything that goes down there (on the West Coast). I have to be open to any situation […] I’ve never not been able to manage to get somebody sorted out within a 24- hour period on the West Coast […]. We’ll just make sure that we’ve got plenty of safety processes in place […] we’ll take orders before I go, we’ll pack them in boxes, and leave them at the backdoors, and money will get handed out in windows […]. (NEP staff)

Being able to “sort someone out” attests to the competencies of peers and the importance of the lived experience workforce. With insider knowledge safe injecting equipment can be provided to PWID in disasters as soon as possible to minimise health risks. Thus, several participants describe having had no problems accessing drug-injecting equipment during the lockdown. For example, Kyle spoke about simply accessing products via the courier service, as arranged by the mobile outreach staff:

It (COVID-19) didn’t affect me at all, systems were in place, and I just used the courier programme via [the outreach staff].

Beyond the practical arrangements provided by the outreach staff, the social and emotional support as part of the peer-based approach to NEPs offers additional resilience for PWID whose lives can be chaotic and changeable. With this extra support being intentional, the mobile outreach staff speaks about providing psychosocial support at every opportunity:

It’s like […] there’s nothing else really solid in their lives a lot of times, but the needle exchange van comes every month, it’s so reliable, and they know I will be there to answer any questions or give whatever support I can. That is the way I’ve worked with this group I’ve given that extra layer of support, every opportunity I can.

Having security or some sense that the world is reliable and stable is important when lives are mostly insecure and uncertain. In the same way, unconditional social and emotional relationality from key support people is important to the wellbeing of minoritised communities. The leadership and camaraderie demonstrated by empathetic NEP staff are valued immensely by the PWID in this study, as reflected in the following quote:

Everyone over here loves and respects the [outreach staff]. He does so much for us over here. Yeah, you’ve just got no idea. (Harriet)

The assertion of “no idea” marks a line in the sand—the intricacies of living as a PWID can only be understood by peers. It also draws attention to the importance of social capital and establishing relationships of trust, whereby PWID can open up to share their needs. Hay and colleagues [7] report that access to peer support at peer-based NEPs is associated with positive indices on anxiety and depression measures, greater satisfaction with life, and increased health-related information exchange with the exchange staff. The strength of these relationships will likely carry over into disasters. This was demonstrated by Kyle when asked how prepared he was for a disaster, he responded that, for him, preparedness included simply having open communication with the outreach staff:

You just gotta have communication about telling [the outreach staff] what’s happening in your life, yeah having communication channels [open], just being prepared.

In Kyle’s narrative, “having communication channels open” shows that reciprocity between PWID clients and NEP staff can lead to information sharing. These findings are like previous studies that suggest the strength of social networks is founded on collaboration, social cohesion and empowerment of people, which can generate resilience during emergencies [76, 77, 78, 79]. Our study also found that more informal peer-based networks, the "key contact" system, within the PWID community was usually an important resource for products and programme accessibility.

#### Key contacts

The PWID community on the West Coast distribute products among themselves, a method used predominantly in remote areas where alternative access options are scarce [10]. It also provides a way of reaching people that might not use the mobile outreach service [24, 10]. The key contact system works by stocking designated PWID members with new injecting equipment to distribute as necessary or during emergencies. Ben explains how the key contacts he knows would have lots of needles if he needed them:

Lots of [key contact distributors] have heaps of needle ends and at the end of the day, if you're worried about it (not having access to needles), say an accident did happen, and the bridge did go down, most would always have a shitload of ends.

Knowing sterile needles were available was reassuring for people like Ben. Due to the uncertainty of the worldwide pandemic, extra precautions were taken to ensure PWID had access to health-sustaining equipment for “two–three months” when it was unsure how the COVID-19 pandemic would unfold and whether NEPs would be classified as essential services. It is important to note that the key contacts were utilised during the 2020 Level 4 lockdown period, and PWID accessed products from them during that time.

The key contacts are reliant on the mobile outreach staff to stock them up, which is a potential limitation. For instance, should road access to Westport be blocked for an extended period of time, access to stock could be difficult. The key contact system is also dependent on amicable interpersonal relationships between members of the PWID community, so some participants expressed concern that conflicts or drug-related dynamics could cause greed, intergroup conflict, and jealousy, limiting the willingness of key contacts to distribute to all members of the community. Ben had experienced gatekeeping by a key contact who appeared selective about whom he shared equipment with. Ben stated: “They say we are a [key] contact, but if we don't like you, we don't want you at our house.”

The potential for gatekeeping, as described by Ben, shows the limitations of the key contact system which, in a sense, mirrors ecological systems where social and material capital enables better response and recovery from a disaster [80, 81]. The mobile outreach staff reported that potential interpersonal conflict between peers could be managed by making PWID on the edge of townships key contacts, or those who are well-liked (have good connections with most). Relational issues more broadly, in the form of stigma, were also seen to interfere with access to NEP products. We now turn to discuss stigma and how that might impact in a disaster.

### Social stigma

Our analysis supported that participants' experiences of stigma played a prominent role in their everyday lives. For the participants, stigma occurred when “respectable citizens” positioned them as inferior, criminals, or immoral, representing a deeply ingrained social mistrust. Some participants described experiences of being ostracised from local sports teams, having limited job opportunities, and even being disowned by family members after they found out about their drug use. The following excerpt represents how stigma remains the greatest struggle in the lives of PWID:

Stigma is our biggest battle by a long way […] we see it happen regularly in small communities. I guess rural New Zealand is a wee bit more judgemental than some of the larger cities. And for a good reason, too, in rural locations, [non-drug users] perceive they have a lot more to lose from the PWID they judge as thieves, unemployable, or always in the courts. (NEP staff)

Stigma is described as a “battle” conjuring warlike imagery, which attests to its deeply traumatising effects. Life is difficult enough for people living in precarity, especially when layered with drug use. Despite that, the participant above demonstrates compassion towards the people who judge them, especially as they might experience some form of harm from the consequences of drug use that is not well understood or supported. These consequences are discussed in more depth in the next section.

#### Stigma as a barrier to other distribution methods

NEP products are also distributed through participating pharmacies; these pharmacies are often community-based and provide a venue for PWID to access NEP products locally, especially as the mobile outreach service only visits the West Coast once per month. The participants in this study were concerned pharmacies might report the purchase of drug-injecting equipment to the local Alcohol and Other Drug Services if they were on managed treatment. This fear acts as a barrier that prevents PWID from using pharmacy-based NEP outlets. Moreover, in less populated areas there can be limited numbers of pharmacies so they dispense both NEP gear and other harm-reduction treatments. The mobile outreach staff believes this is problematic:

[Our area] only has one pharmacy, it does NEP, and it does OST, so there's a real dilemma for some PWID asking for injecting equipment from the people that are distributing their methadone, who take a personal view on this and say, "You shouldn't be injecting your methadone", when, once it goes out to the shop, it's down to the PWID what they do with it.

The experience of discrimination described in this excerpt comes from the misconception that injecting methadone is illegal and other moral discourses that say some mood-altering substances (like alcohol) are socially acceptable when others are not. It is also founded on substance gestation; it is tolerable to consume methadone orally, but not inject it:

It's not actually illegal to inject, and that's what a lot of confusion is about for a lot of people that don't know a lot about our world. […] we've had phone calls from people saying, "I've just come out of the pharmacy to pick up some injecting equipment, and they just looked at me like a piece of dirt". That doesn't help any process in any way, shape or form, you know? […] ‘cos to keep 'em (PWID) safe, they need that new equipment. You stigmatise, they might not come back for a week and go home and (re-)use that same pre-used syringe and needle.

While health services might have a duty of care, as the mobile outreach staff suggests above, healthcare services should prioritise the health and wellbeing of PWID. The goal of harm-reduction is to prevent harms associated with injecting and the aim of opioid treatment is to mitigate the chronic and relapsing pattern of problematic illicit opioid use, making it necessary to not promote abstinence to the exclusion of substance-using harm reduction methods [82]. The mobile outreach staff point to the irony of stigmatising PWID for trying to keep themselves safe. Participants also note the role of stigma in broader emergency management practices, which we now turn to discuss.

#### Fear of mistreatment from emergency managers

When a large-scale disaster strikes, emergency management personnel respond to community needs which can include setting up community welfare centres for evacuations or providing support (e.g. information, food, psychosocial care) [83]. As noted, the participants in this study express fear that they would be deprioritised by emergency management staff in such a setting. In the following excerpt, Finn reflected on how his “drug-using status” could impact the way he would be treated:

[Emergency responders] will be saying, “look, you're getting so many drugs that you could knock out a horse, you’re the last people we should take care of, you put yourself in that situation” […]. I just think out of everything they'd think of; no one would even think about all these guys are going without their meds. It's more like, oh well, the druggies are going to have to sit back for three days and handle it.

Alongside Finn, other participants also felt that they would not be supported, and instead be considered undeserving. This anticipated stigma is borne from the way in which they have been treated in other areas of their lives. The mobile outreach staff described experiences of inequitable treatment and general social scorn:

[PWID] know they're not getting treated the same as others are getting treated. Especially around the prescribing of their drugs […]. I was listening to the radio the second week of lockdown, and I heard one guy come on, and he said,” I know how to sort all the junkies through this period! You just get rid of drugs, and you put them all in lockdown - you put them all in prison. You've got to put all the addicts in prison”. What? Why would anyone ever think something like that! […] Sometimes, we prove them right, but often we prove them completely wrong.

In his narrative, the staff member also states,”everybody is lax around our people”, meaning the care and attention that PWID need to manage their problems are ignored. Knowing social systems produce a bias towards them and encountering stigmatising attitudes can be distressing for PWID. Stigma can prevent them from seeking help or acting defensively when engaging with the general public. Interviewing people on OST in Aotearoa New Zealand [41], similarly found participants anticipated they would be disregarded in disaster situations by emergency management personnel.

### Solutions for accessibility

Given the health risks when NEP products are unavailable, the participants in this study offered a range of solutions for equipment distribution during an emergency. These included collaborating with local emergency personnel, storing injecting equipment for emergencies in community spaces, providing free drug-injecting equipment, and installing electronic dispensers.

The mobile outreach staff was optimistic about collaborating with emergency managers or key people in the community to store sterile drug-injecting products in various locations along the West Coast of the South Island:

Wouldn't it be great if we get some injecting products stored away in [Westport, Hokitika and Greymouth], maybe in community halls? We have all these community halls right around New Zealand in the smallest of areas, like where there are only two-three hundred people, there is a community hall!

This participant had spoken to a civil defence representative with “a fantastic attitude towards PWID”, who had suggested community halls as a space to store sterile drug-injecting equipment. This kind of alliance is imperative to the welfare of those who use drugs in rural communities, especially in small towns, because it could overcome travel barriers; however, storing injecting equipment in public venues would need careful planning and community buy-in.

Another participant, David, thought that using community halls would still be challenging in a disaster, given the long distance between townships:

There is 111 kilometres between [Hokitika and Greymouth], so even in a weather event, we can't get to Westport to get needles […]. We should only have [emergency NEP equipment] on this side of the bridge.

In this excerpt, David suggests alongside travel issues, critical infrastructure vulnerabilities like bridge collapses due to floodwaters present additional risks. This is a real concern for people living on the West Coast, as evidenced by a 2019 flood that washed away part of a bridge [84]. Others added that travelling too far also presents financial barriers, Alice highlights "there is no way" people would drive to the neighbouring towns to pick up products because of the cost of transport or petrol. Along with earlier indications of user-pay products costing too much, this strengthens the idea that solutions should be local and free to maximise accessibility for PWID. David suggested free equipment as a solution during an emergency:

I think if there was an emergency, [equipment] would have to be free, ‘cos you wouldn't have the money […] it's just that money machines would be out, and electricity could be gone. You know anything could happen.

Contrary to free equipment and travel costs, a second popular solution identified by participants was electronic dispensers. Electronic dispensers (vending machines) already exist in all static NEP services in Aotearoa New Zealand. Vending machines are stocked with user-pays products (for instance: one syringe, one needle, one filter) and cost between 2 and 5 dollars (NZD). There are no vending machines on the West Coast or any other remote areas in Aotearoa New Zealand. Participants argued that installing one in the main West Coast towns (e.g. Westport, Hokitika, Greymouth) would provide a backup service for accessing NEP products in situations where the mobile outreach service cannot travel into the respective areas. However, it is important to note that they do also require restocking which could be problematic during a disaster. It further dissipates any potential barriers associated with interpersonal issues, including jealousy or favouritism with key contact distribution. No one can be a gatekeeper with a vending machine, as indicated by Alice:

No one is in control with that, even though behind the scenes [someone] might be filling them.

Vending machines, unlike the NEP-based pharmacies, would lessen the possibility of being stigmatised by non-drug users or the public. However, vending machines still pose serious confidentiality risk in small areas where everyone knows everyone, and “gossip” is common:

Just the fear of being [seen] going to that machine could jeopardise a lot of people's employment... big gossip town, mate. (Max)

Despite the risk to people's anonymity, both storing equipment in community venues and installing vending machines were favoured local solutions that could overcome critical infrastructure disruptions in disaster situations. Overall, these findings suggest that maximising accessibility to equipment involves locally accessible equipment as vital to emergency management planning, ideally delivered in a way that protects the identity of PWID to avoid stigma and minimise the potential consequences of being recognised as a PWID.

## Conclusion

While barriers to healthcare for PWID are documented well in the international literature, to our knowledge, this was the first study to map access vulnerabilities for PWID living in a rural area of Aotearoa New Zealand. This study aimed to understand what enables and constrains access to NEP services on the West Coast of the South Island in Aotearoa New Zealand during extraordinary times. Our findings were consistent with previous literature on service accessibility.

Notably, access to healthcare was recognised as especially difficult for people living in remote regions, characterised by limited access or poor infrastructure and smaller populations [56]. Correspondingly, we found that road network disruptions, distance, and a lack of local infrastructure were service continuity concerns for the NEP mobile outreach service during natural hazards, disasters, and, in fact, transpired during the early stages of the 2020 COVID-19 lockdown in Aotearoa New Zealand when travel restrictions were in place. Also, evident where financial barriers [12, 15] even though NEP products are already subsidised in Aotearoa New Zealand. Hardship is an everyday lived experience for some PWID, and the prevailing disadvantages are often intensified in a disaster [85, 41, 86, 87]. Cost will hinder drug-injecting equipment accessibility. Social stigma during disasters was also a major concern for PWID in this study, as found in other research [20, 88, 89, 11]. Fear of repercussions from healthcare professionals and OST services could prevent participants from picking up equipment at a public location and produced fear that they would be ignored in emergency management response and recovery efforts.

Salient to our findings and indicative of broader disaster management research was the value of social capital, the networks and connections between people that are associated with good disaster resilience for rural communities [56]. This study reinforced the importance of peer-based relational systems, both, formal roles (the mobile outreach staff) that arranged resources via networks and informally (as key contacts), for material and social resilience.

These findings have implications for NEP in emergencies, as well as broader emergency management. A range of potential solutions to accessibility concerns arose, such as liaising with emergency management personnel, finding safe community spaces to store drug-injecting equipment and ensuring access to cost-free equipment. Another possibility, as exists in static NEP exchanges, are electronic dispensers. While this option has set up costs, the long-term payoffs for health and wellbeing for the PWID community would surpass the initial outlay.

We suggest that PWID be empowered to collaborate with health authorities and emergency management personnel to maximise local backup disaster plans. Importantly, this requires a paradigm shift away from the taken-for-granted "one-size-fits-all" approach to emergency management largely practised in Aotearoa New Zealand. It is necessary to replace broad population-based strategies and attend to the specific preparedness needs of minoritised groups to enable targeted disaster and health care practices [42, 66].

Research exploring the disaster management needs of remote and minoritised communities in nations like Aotearoa New Zealand is scarce and only beginning to be undertaken [90, 91]; moreover, of the studies so far, none have focused on PWID. Locally tailored protocols and guidelines are needed to encourage service continuity for NEP in disasters [92], particularly given the ongoing threats of natural hazards and human-generated events in times of Covid-19 and the Anthropocene. Ongoing research is necessary to continue to explore the views and experiences of PWID to garner how best to support them, on their terms, during disasters. Future research could address solutions that support PWID to stock up on essential health-sustaining equipment and other preparedness items to minimise physical contact with others, and this should be considered within the context of natural hazard preparedness. PWID are a minoritised group that face health risk and access vulnerabilities, particularly when living in rural areas. These services should be supported to have adaptive capacity [93] in extraordinary times where additional stressors are induced. Substantial financial support from central and local governments [9] would render social support and empower PWID and others who experience hardship to be safe when the world is unstable and insecure.

## Data Availability

The datasets generated and/or analysed during the current study are not publicly available due the sensitivity of the material, but are available from the corresponding author on reasonable request.

## Abbreviations

NEP: Needle Exchange Programme
PWID: People who inject drugs
NZNEP: New Zealand Needle Exchange Programme
HIV/AIDs: Human immunodeficiency virus/acquired immunodeficiency syndrome
HBV: Hepatitis B virus
HCV: Hepatitis C virus
1–4-1: One-for-one
OST: Opioid substitution treatment
IPA: Interpretive phenomenological analysis
NEMA: National Emergency Management Agency
COVID-19: Coronavirus disease
NZC: New Zealand dollars

## Footnotes

1 While this is a commonly accepted acronym, the authors recognise that not all people who inject drugs like how this term represents them.

2 The term drugs in this article refer to illicit drugs and/or  Opioid Substitution Treatment.

3 All names used in this research are pseudonyms
